# Effect of Perillaldehyde on Prophylaxis and Treatment of Vaginal Candidiasis in a Murine Model

**DOI:** 10.3389/fmicb.2019.01466

**Published:** 2019-07-02

**Authors:** Su Qu, Lei Chen, Hui Tian, Zhen Wang, Fei Wang, Liqin Wang, Jinting Li, Hui Ji, Liurong Xi, Zhaojun Feng, Jun Tian, Zhaozhong Feng

**Affiliations:** ^1^College of Life Science, Jiangsu Normal University, Xuzhou, China; ^2^Beijing Advanced Innovation Center for Food Nutrition and Human Health, Beijing Technology and Business University, Beijing, China

**Keywords:** *Candida albicans*, perillaldehyde, antifungal, vaginal candidiasis, murine model

## Abstract

Vulvovaginal candidiasis is a common fungal infection afflicting women which is primarily caused by the yeast *Candida albicans* (*C. albicans*). It is imperative to introduce new drug classes to counter this threat due to the continuous emergence of drug-resistant cases in recent years. The purpose of this study was to clarify the *in vivo* antifungal activity of perillaldehyde (PAE) against *C. albicans* and to prove that PAE is a promising candidate for the control of vaginal candidiasis. An animal model of vaginitis was developed to demonstrate the therapeutic and preventive effects of PAE on vaginal candidiasis, and these were evaluated through fungal and histopathological examinations. In clarifying the mechanism of PAE, standard hematological test results indicated that white blood cells (WBC) were elevated abnormally in mice infected with *C. albicans*, whereas when the mice were treated with various concentrations of PAE, the number of WBC in the blood was reduced. Flow cytometry was used to detect the populations of neutrophils, macrophages and CD4 T cells in the vaginal tissue of the mice. PAE was found to reduce these immune cells, which all play a key role in the inflammatory response, and the related interleukin and pro-inflammatory cytokines, including IL-17, IL-22 and TNF-α. These were detected using ELISA. Finally, we detected the expression levels of E-cadherin in the PAE treatment mouse group and discovered that it had recovered to its normal levels, but in the infection mouse group, the E-cadherin expression was clearly suppressed by the presence of *C. albicans*. Our data demonstrated that PAE targets these cytokines and possesses the ability to fight the fungal infection while also reducing the levels of the inflammatory factors identified. Our results demonstrated that PAE has a significant preventative and therapeutic effect on vaginal candidiasis and is a potential candidate for the treatment of vaginal *Candida* infections.

## Introduction

Invasive candidiasis is an opportunistic infection caused by the *Candida* fungal species. *C. albicans* is a commensal yeast found in the skin, mucous membranes, gastrointestinal tract, blood and vagina of animals and humans (De Luca et al., 2013). However, when the human immunity is diminished or the internal microecological environment is changed, recurrent mucosal *Candida* infections may occur, and even an invasive candidiasis infection is possible (Bondaryk et al., 2013). When considering the large number of infections acquired within the hospital setting, infection by *Candida* is the fourth highest, with a death rate as high as 40% (Lee et al., 2009). Vulvovaginal candidiasis (VVC) and recurrent vulvovaginal candidiasis (RVVC) represents a continual and universal crisis, which torments 70 to 75% of women, worldwide, at least once in their lifetimes. The reasons for the rapid multiplication of VVC and RVVC include the fact that they are caused by *C. glabrata*, *C. tropicalis, C. albicans*, and atypical *C. albicans* such as *C. africania*, but of these, *C. albicans* predominates, occurring in the vaginas of 90% of patients (Trama et al., 2005; Yazdanparast et al., 2015). Thus, the polymorphic microorganism *C. albicans* is a normal and opportunistic resident in the vagina and the major causative agent of VVC (Achkar and Fries, 2010). The ever-increasing incidence of vaginal candidiasis seriously affects women’s work and personal lives. Patients with chronic and recurring attacks suffer from tremendous discomfort, which is both physiological and mental (Sobel, 2016). The effectiveness and the toxicity levels of the drugs used to target *C. albicans* needs to be explored so that women’s VVC can be treated effectively and prevented from impacting their daily lives.

Currently, the drugs used for the clinical treatment of vaginal candidiasis are normal chemical compounds such as polyenes (amphotericin B), azoles (fluconazole), and echinocandins (caspofungin). Although these medicines are generally effective at remitting the disease burden and its symptoms, all these drugs contribute to varying degrees of drug resistance in the course of treating the vaginal candidiasis because of the static function of the drugs and the microorganism recalcitrance (Sobel et al., 2003; Pappas et al., 2016). Furthermore, these antifungal drugs all have disadvantages in terms of their activity profiles, their pharmacokinetic properties, and their host toxicity, indicating the urgent need to discover new types of antifungal compounds (Campoy and Adrio, 2017).

For some decades, researchers have been interested in the antifungal effects of essential oils in natural products, and obvious effects have been achieved using antifungal drugs developed from medicinal plants since early in the 20th century (Pinto et al., 2013). Perillaldehyde (PAE) is a natural monoterpene compound occurring abundantly in perilla, a medicinal herb of the mint family. PAE is a colorless to pale yellow liquid with a variety of biological properties, including anti-inflammatory, anti-oxidative, anti-allergic, anti-bacterial, and the effect of a reduction of fat in the blood (Ahmed, 2018; Fuyuno et al., 2018; Uemura et al., 2018). The preliminary work done by our team has shown that PAE demonstrates significant antifungal activity against filamentous fungi such as *Aspergillus flavus* and *Aspergillus niger* (Tian et al., 2015, 2016). Recently, our group has also shown that PAE has a remarkable antifungal effect on *C. albicans*, with a minimum inhibitory concentration (MIC) of 0.4 μL/mL (Tian et al., 2017b). The team has also identified that the effect of PAE on *C. albicans* occurs through the mechanism of Ca^2+^ and oxidative stress-mediated apoptosis (Tian et al., 2017a). However, no research reported in the literature has been designed to investigate the application of PAE to the treatment of *C. albicans* vaginitis. Furthermore, the effects of using a PAE vaginal cleaning fluid frequently, have not been evaluated, thus there are no reports to show whether regular use of PAE can protect the vagina in advance.

For this investigation, which was based on the antifungal activity of PAE against *C. albicans* as evident *in vitro*, a murine model of VVC was established. This model would enable the preventive and therapeutic effects of PAE to be evaluated *in vivo*. Furthermore, hematology and cytokines would be investigated because they are known to play a crucial role in immunity and inflammatory disease, and it was still unclear whether PAE targets these factors (Conti et al., 2009; Wang W. et al., 2016). This research aimed to solve these issues and to provide a reliable reference for the use of PAE as a safe, effective and practical drug candidate for both treatment and prevention in women suffering from VVC and RVVC.

## Materials and Methods

### Materials

Perillaldehyde (CAS registry no. 18031-40-8) was obtained from the TCI Development Co., Ltd. (Tokyo, Japan), prepared as a stock solution in 0.1% (*v/v*) Tween-80. Fluconazole and estradiol benzoate oil were acquired from Solarbio Science & Technology Co., Ltd. (Beijing, China).

### *Candida albicans* Strain and Growth Conditions

The *C. albicans* ATCC 64547 used in this study was purchased from the American Type Culture Collection (ATCC) (Manassas, VA, United States). The strain to be tested was stored in frozen stock with 15% glycerol at −80°C. Prior to each experiment, the *C. albicans* was freshly inoculated onto sabouraud dextrose agar (SDA) to ensure optimal growth characteristics and purity.

### Experimental Animals

Six-week-old female BALB/c (22 ± 2 g) mice were purchased from Beijing HuaFuKang Bioscience Co., Inc. (Beijing, China) for all the animal experiments. Before the experimental stage, all mice were fed in the IVC system for 1 week and kept under controlled conditions at a temperature of 22–24°C, at a relative humidity of 60% and in a 12/12-h light/dark cycle. All mice had free to access to water and food. All animal experiments complied with Chinese legislation on the use and care of experimental animals. The agreement was authorized by the animal experiment committee of Jiangsu Normal University (No: 201703003; March 6, 2017) and ensured that the pain of the experimental animals was minimized.

### Construction of an Animal Model of Vaginitis

The model of vaginitis was established according to previous reports (Munoz et al., 2017) with some modifications. The estradiol benzoate was dissolved in sesame oil and ultrasonic to promote its dissolving. PAE and fluconazole (FCZ) were diluted in 0.01% Tween-80 to 0.4, 0.8, and 1.6 μL/mL. The mice were injected subcutaneously with 0.2 mg of estradiol benzoate every other day to a total of three times in the week. The mice were then anesthetized with 1.25 mg/kg urethane and were inoculated into the vaginal lumen with 20 μL of 6 × 10^8^ cells/mL *C. albicans* spores. The day after the murine model was accomplished, 500 μL PBS was used on the mice to perform vaginal lavage and the fungal burden in the lavage fluid was assessed under a microscope to ensure that each mouse was infected with *C. albicans*. The prevention and treatment group mice were then treated with either 0.77, 1.54, 3.08 mg/kg PAE or 20 mg/kg FCZ. All mice were killed on day five.

### Preventive Effect

Mice in the preventive effect experimental group were randomly divided into six groups (*n* = 10) after 1 week-adaptable feeding. In the preventive experiment, the administration of the different concentrations of PAE and fluconazole (FCZ) began 2 days prior to the vaginal inoculation with *C. albicans.* The P-WT group was given 0.1% (*v/v*) sterile Tween-80 every day and these mice were not infected with *C. albicans*, but this group was given the same dose of anesthetic. The P-Ca group was given the same dose of 0.1% (*v/v*) sterile Tween-80 and anesthetic and it was infected with *C. albicans*. The P-0.77 mg/kg PAE group was given PAE at a concentration of 0.77 mg/kg, and the P-1.54 mg/kg PAE group was given PAE at a concentration of 1.54 mg/kg, while the P-3.08 mg/kg PAE group was given 3.08 mg/kg PAE. The P-20 mg/kg FCZ group was given Fluconazole at a concentration of 20 mg/kg. Each time, 20 μL of all drugs were given, and they were administered twice daily. The administration of the PAE and fluconazole continued for 5 days.

### Therapeutic Effect

As in the preventive experiment, in the therapeutic experiment there was a control group of mice that were not infected with *C. albicans* and five groups of mice who were infected with *C. albicans* after 1 week-adaptable feeding. The T-WT group was given 0.1% (*v/v*) sterile Tween-80 every day, they were not infected with *C. albicans*, but this group was given the same dose of anesthetic. The T-Ca group was given the same dose of 0.1% (*v/v*) sterile Tween-80 and the anesthetic, and it was infected with *C. albicans*. All other groups were infected with *C. albicans* before their treatment started: The T-0.77 mg/kg PAE group was given PAE at a concentration of 0.77 mg/kg, and the T-1.54 mg/kg PAE group was given PAE at a concentration of 1.54 mg/kg, while the T-3.08 mg/kg PAE group was given 3.08 mg/kg PAE. The T-20 mg/kg FCZ group received fluconazole at a concentration of 20 mg/kg (Gao et al., 2016). Each time, 20 μL of all drugs were given and they were administered twice daily. The administration of the PAE and fluconazole continued for 5 days.

### Fungal Test

We measured the amount of fungus in the vaginas of the mice by counting the colony forming unit (CFU). All mice were sacrificed 5 days after the vaginal infection and the vaginas were removed and weighed. The tissues were thoroughly blended in a homogenizing apparatus with 1 mL of PBS (Munoz et al., 2017), and then 100 μL of the homogenate was inoculated into a Candida chromogenic medium (Hopebio, China). Colonies were counted visually after 48 h of incubation at 30°C.

### Histopathological Examination

The mice were sacrificed 5 days after being infected, and the tissue was transferred to 4% paraformaldehyde (pH 7.4) and fixed at 4°C for 4 h. The tissues were then held at 4°C in a 30% (*v/v*) dehydration solution overnight and then embedded in a compound at an optimal cutting temperature. Finally, 12 μm sections were selected for the frozen section machine (Leica, Germany) and for staining with Periodic Acid-Schiff (PAS) and hematoxylin-eosin (H&E). Under the microscope, at 10× and 40× magnification, we could observe the formation of mycelium and the changes in histomorphology that followed the *C. albicans* infection.

### Determination of Hematological Indexes in Mice

Each mouse delivered 300 μL of blood into the anticoagulant tube. The white blood cell (WBC) counts, red blood cell (RBC) counts (along with associated variables, such as hemoglobin [HGB], red blood cell specific volume [HCT], erythrocyte mean corpuscular volume [MCV], mean corpuscular hemoglobin [MCH], mean corpuscular hemoglobin concentration [MCHC], and red cell volume distribution width [RDW-CV and RDW-SD]), and blood platelet (PLT) counts (along with associated variables, such as mean platelet volume [MPV], platelet distribution width [PDW] and PCT) were then all measured by a three-class automatic blood-cell counter (Mindray, China).

### Detection of Immune Cells

The tissues were cut and homogenized in a homogenizing apparatus and 10 ml sterile saline was added to prepare a cell suspension that was filtered through a 200-micron nylon mesh. The cell suspension was centrifuged at 1500 × *g* for 5 min, and then the supernatant was discarded. The precipitate was resuspended by adding red blood cell lysate and was allowed to stand at room temperature for 5 min. After neutralization of the cell suspension with PBS, centrifugation was performed at 1500 × *g* for another 5 min, and then the supernatant was discarded. The cell suspension was then washed and resuspended once again with PBS and then incubated with the rat anti-mouse CD4-FITC, F4/80-PE, Ly6G and LY-6C-FITC (BD Biosciences, United States) for 30 min on ice. Finally, the cells were analyzed by flow cytometry (BD Biosciences, United States).

### Measurement of Cytokines

The tissues were homogenized with a protease inhibitor and red blood cell lysate, and samples were centrifuged at 5000 × *g* for 10 min. The supernatant was taken and stored at −80°C. The IL-17, IL-22, and TNF-α levels in the solution were measured with an enzyme-linked immunosorbent assay (ELISA) kit (Elabscience, China). The OD value was measured with an enzyme-labeled instrument (Thermo Fisher, United States).

### Western Blot Analysis

The vaginal tissue was homogenized in lysis buffer andcentrifuged at 13000 rpm for 30 min at 4°C, the supernatantwas then pipetted gently into a new tube. Total protein wasquantified by the BCA Protein Assay Kit (Solarbio, China) and boiledat 99°C for 8 min. The sample was separated by SDS-PAGE(6%) and transferred to a polyvinylidene fluoride (PVDF) membrane(Merck Millipore, United States). The PVDF membrane was blocked with5% non-fat milk (m/v) for 1 h and then washed with 0.1% Tween-20 inTris saline buffer. It was then incubated with rabbitanti-E-cadherin/HARP (Bioss, China) at 4°C overnight. Thepresence of E-cadherin on the membrane was detected withWestern Blot chemiluminescence reagents (GE Healthcare, United States), and the reactive density was measured with Image Jsoftware 1.48 V.

### Statistical Analysis

Data were expressed as mean ± standard error (*n* = 6). The data were all evaluated by the Kruskal–Wallis test as is appropriate when using the GraphPad Prism software, version 8.0.0. Statistical significance was set at *p* < 0.05 for all tests.

## Results

### PAE Was Effective With Vaginal *Candida in vivo*

**FIGURE 1:**
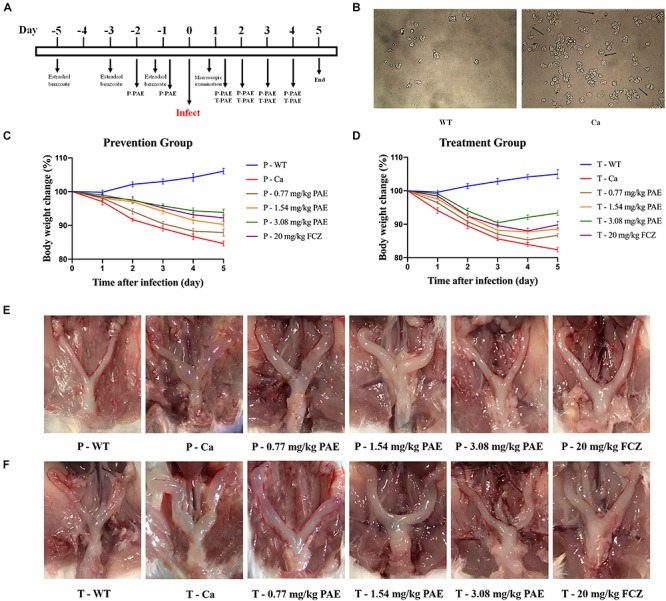
Murine model of vaginal candidiasis and its features. **(A)** Timeline of *C. albicans* infection and PAE treatment of model. **(B)** Verification of *C. albicans* infection: black arrows indicate the hyphal of *C. albicans* in vaginal lavage fluid. **(C,D)** Mice body weight changes during the vaginal infection process. **(E,F)** Macroscopic images showing the inflammatory levels in uterine tissue taken from each group.

We evaluated the efficacy of PAE *in vivo* by using an experimental murine model for vaginal candidiasis (Figure 1A). As shown in Figure 1B, the mouse model evidenced a significant mycelial phase of *C. albicans*, as was seen by microscopic examination. This indicated that the mice had been successfully infected with *C. albicans*, while no mice died. In the prevention and treatment experiments, our data showed that mice infected with *C. albicans* lost weight compared with the WT mice, but the difference diminished with an increase in the concentration of PAE. The change in body weight was also less in the P-3.08 mg/kg PAE and T-3.08 mg/kg PAE groups than in the P-20 mg/kg FCZ and T-20 mg/kg FCZ groups (Figures 1C,D). Interestingly, we found that uterine inflammation was significantly relieved by the PAE prevention and treatment (Figures 1E,F), while the fur of mice in the Ca group exhibited a dull yellow, uneven color, and their coats were less supple than the coats of the WT and 3.08 mg/kg PAE groups. Weight loss values were consistent with the recovery from uterine inflammation.

### PAE Reduced the Amount of *C. albicans* in the Vaginal Canal

**FIGURE 2:**
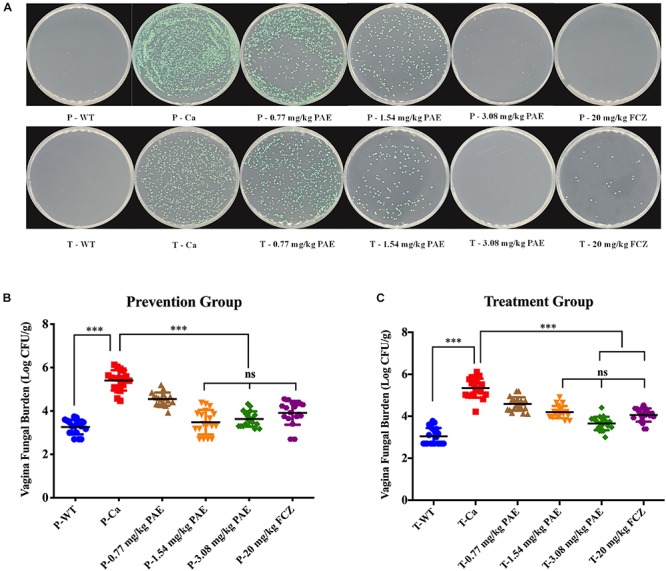
PAE reduces the vaginal fungal burden (log CFUs/g) in different groups of mice. **(A)** The amount of *C. albicans* in VVC mice was quantified by using a plate count, the vaginal homogenate was used to coat the candida chromogenic medium. The *C. albicans* colonies were shown as green in color. **(B)** The CFU count in the PAE prevention group. ^∗∗∗^ means *p* < 0.001, ns means not significant, error bars, ± SD. **(C)** The CFU count in the PAE treatment group. ^∗∗∗^ means *p* < 0.001, ns means not significant, error bars, ± SD.

To better illustrate the dosing with PAE, we quantitated the amount of fungus in the vaginal canal by CFU counting. After being cultured at 30°C for 48 h, the color of *C. albicans* showed as a green color, *C. tropicalis* showed as blue-gray, *C. glabrata* showed as lavender to purple, *C. clostridia* showed as purple-pink, and other Candidiasis showed as white. In Figure 2A, the plate shows mostly *C. albicans* and no other Candida. Assuming this was related to the inoculation of *C. albicans* into the mouse model, we performed the CFU counts on the green fungi. There was a significant difference between the WT group and the Ca group (*p* < 0.001), and the 3.08 mg/kg PAE and 20 mg/kg FCZ groups were also significantly different from the Ca group (*P* < 0.001). In addition, the high doses of PAE and the fluconazole were shown to be equally effective (*P* < 0.05) (Figure 2C). In the prevention experiment, we found a trend similar to that in the treatment, except that in the prevention experiment 1.54 mg/kg PAE had the same effect as the higher doses of PAE and fluconazole in the treatment group (Figure 2B). Therefore, 3.08 mg/kg PAE may be the optimal dose for the prevention and treatment of vaginal candidiasis in the murine model.

### PAE Reduced the Vaginal Fungal Burden and Inflammation

**FIGURE 3:**
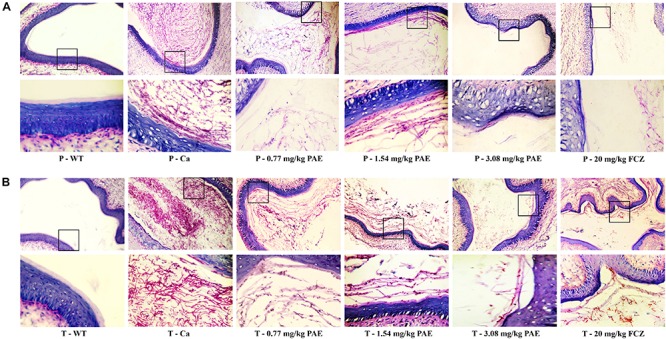
Histological evaluation of vaginal *C. albicans* invasion. **(A)** Mice in the prevention group were first injected with estrogen. They were then given either 0.77, 1.54, 3.08 mg/kg PAE or 20 mg/kg FCZ to act as a preventative. Two days later, they were infected vaginally with *C. albicans*. After 5 days, their tissues were harvested, and sections stained with PAS. The results were observed under 10× and 40× magnification. The red filaments in vaginal tissue were *C. albicans* hyphae. **(B)** Mice in the treatment group were treated with estrogen and then infected vaginally with *C. albicans*. These infected mice were then treated with either 0.77, 1.54, 3.08 mg/kg PAE or 20 mg/kg FCZ. Their tissues were harvested, sectioned and stained with PAS. The results were observed under 10× and 40× magnification. The red filaments observed in the vaginal tissue were *C. albicans* hyphae.

**FIGURE 4:**
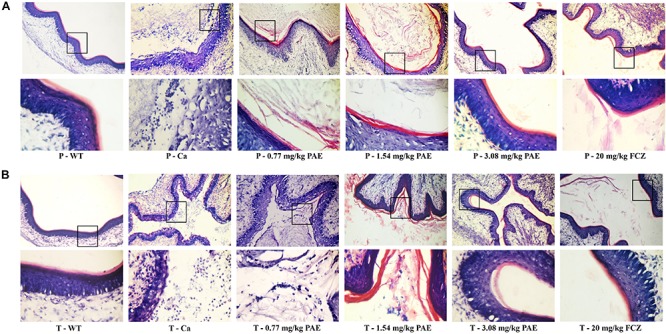
Histological evaluation of vaginal damage and inflammatory levels. **(A)** Mice in the prevention group were injected with estrogen and then 0.77, 1.54, 3.08 mg/kg PAE or 20 mg/kg FCZ was given as a preventative treatment 2 days prior to the mice being infected vaginally with *C. albicans*. Five days later, the tissues were harvested and sectioned for HE staining. The results were observed under 10× and 40× magnification. The little blue and purple dots indicate PMNs. **(B)** Mice in the treatment group were treated with estrogen and then infected vaginally with *C. albicans*. The infected mice were then treated with 0.77, 1.54, 3.08 mg/kg PAE or 20 mg/kg FCZ. After 5 days, the tissues were harvested and sectioned for HE staining. The results were observed under 10 × and 40× magnification. The little blue and purple dots indicate PMNs.

In order to further understand the protective effect of PAE on the vagina, we evaluated the extent of candidiasis by fungal load and histology in both the prevention and treatment experiments 5 days after setting up the mouse model (Figures 3, 4). Microscopic analysis showed that *C. albicans* invaded the keratin and destroyed the overall structure of the keratin. In contrast, mice treated with PAE exhibited only superficial mycelial invasion and an intact tissue structure. The effects of 3.08 mg/kg PAE and 20 mg/kg FCZ were most pronounced, and in these mice, *C. albicans* hardly invaded the keratin (Figure 3B). In addition, those mice with no *C. albicans* were shown to have intact lamina propria, squamous epithelium and keratin. In sharp contrast to the uninfected mice, the vaginal tissue of mice infected with *C. albicans* showed significant inflammation and that a large number of polymorphonuclear neutrophils (PMNs) had been recruited. After the 0.77 and 1.54 mg/kg PAE treatments, the vaginal tissues of the mice were significantly improved and the PMNs were reduced, indicating a certain ability for repair. At doses of 3.08 mg/kg PAE and 20 mg/kg FCZ, the treatment group were almost completely resistant to the *C. albicans* infection (Figure 4B). In the prevention group, we found that PAE had a slightly better protective effect on the vagina than in the treatment group, and there was a distinct alleviation of the effect of the *C. albicans* invasion (Figures 3A, 4A). H&E and PAS staining demonstrated that PAE can effectively prevent and treat vaginal candidiasis, which is consistent with our previous visual observations.

### Hematological Evaluation of the Therapeutic Mechanism of PAE

The hematological parameter is a virtual reference for inflammation, and it is widely used in immunology, medical genetics, oncology, blood banking, transfusion medicine, clinical pathology, and laboratory medicine (Coller, 2015). Our hematological results indicated that WBCs, red blood cells, hemoglobin and hematocrit all exhibited obvious changes in the Ca group compared with the WT group. Gradually, these data were all reduced to a normal level, and the mice in the FCZ treated group also indicated a better response to treatment (Table 1). However, when these four indicators were removed, all other parameters showed no change. Finally, the data for the prevention group were all slightly better than those for the treatment group (Table 2).

**TABLE 1:** Hematology analysis in the prevention group.

	**WBC**	**RBC**	**HGB**	**HCT**	**MCV**	**MCH**	**MCHC**	**RDW-CV**	**RDW-SD**	**PLT**	**MPV**	**PDW**	**PCT**
	**(10 ^9^/L)**	**(10 ^12^/L)**	**(g/L)**	**(%)**	**(fL)**	**(pg)**	**(g/L)**	**(%)**	**(fL)**	**(10^9^/L)**	**(fL)**	**(%)**	**(%)**
P-WT	3.31 ± 1.40	8.42 ± 0.26	129.50 ± 1.87	37.83 ± 0.70	50.17 ± 0.75	16.98 ± 0.22	341.50 ± 4.28	13.30 ± 0.51	28.32 ± 0.58	231.33 ± 70.45	6.17 ± 0.36	15.17 ± 0.54	0.05 ± 0.02
P-Ca	6.25 ± 0.72^a^	7.85 ± 0.33^a^	140.50 ± 2.26^b^	41.10 ± 1.40^b^	50.30 ± 0.36	16.95 ± 0.24	340.50 ± 5.01	13.12 ± 0.38	27.68 ± 0.56	170.67 ± 68.21	6.18 ± 0.39	15.18 ± 0.29	0.11 ± 0.08
P-0.77 mg/kg PAE	4.43 ± 1.80	8.06 ± 0.31	139.50 ± 2.43^a^	39.78 ± 0.77	50.37 ± 0.87	16.92 ± 0.28	340.83 ± 2.86	13.33 ± 0.47	28.80 ± 1.01	230.33 ± 139.30	6.18 ± 0.32	15.15 ± 0.48	0.13 ± 0.11
P-1.54 mg/kg PAE	3.66 ± 0.82	8.11 ± 0.26	136.67 ± 5.13	39.27 ± 0.99	50.37 ± 0.77	17.15 ± 0.35	340.50 ± 4.46	13.23 ± 0.40	28.28 ± 0.47	198.00 ± 141.23	6.10 ± 0.26	14.97 ± 0.33	0.08 ± 0.04
P-3.08 mg/kg PAE	3.04 ± 1.21	8.33 ± 0.44	132.17 ± 3.60	38.05 ± 0.65	50.02 ± 0.74	17.02 ± 0.29	340.67 ± 2.66	13.20 ± 0.36	28.48 ± 0.42	233.50 ± 146.25	5.98 ± 0.26	15.05 ± 0.43	0.01 ± 0.08
P-20 mg/kg FCZ	3.42 ± 0.74	8.16 ± 0.23	133.83 ± 5.42	38.97 ± 0.93	50.00 ± 0.64	17.07 ± 0.34	340.67 ± 2.34	13.27 ± 0.29	27.80 ± 0.85	258.17 ± 182.89	6.10 ± 0.38	15.13 ± 0.35	0.09 ± 0.05

*^*a*^means compared with the WT group: *p* < 0.05, ^*b*^means compared with the WT group: *p* < 0.01, and ^*c*^means compared with the WT group: *p* < 0.01.*

**TABLE 2:** Hematology analysis in the treatment group.

	**WBC**	**RBC**	**HGB**	**HCT**	**MCV**	**MCH**	**MCHC**	**RDW-CV**	**RDW-SD**	**PLT**	**MPV**	**PDW**	**PCT**
	**(10 ^9^/L)**	**(10 ^12^/L)**	**(g/L)**	**(%)**	**(fL)**	**(pg)**	**(g/L)**	**(%)**	**(fL)**	**(10^9^/L)**	**(fL)**	**(%)**	**(%)**
T-WT	3.22 ± 1.02	7.66 ± 0.19	128.67 ± 2.16	37.40 ± 0.42	49.62 ± 0.17	17.15 ± 0.15	344.00 ± 3.79	13.33 ± 0.12	27.42 ± 0.10	165.50 ± 69.69	6.03 ± 0.37	15.20 ± 0.32	0.10 ± 0.03
T-Ca	6.10 ± 0.27^b^	8.43 ± 0.23^b^	141.17 ± 3.49^b^	41.98 ± 0.34^c^	51.17 ± 0.31^b^	16.65 ± 0.14	340.17 ± 3.37	12.95 ± 0.08	26.57 ± 0.29	270.67 ± 189.29	6.10 ± 0.40	15.07 ± 0.27	0.13 ± 0.11
T-0.77 mg/kg PAE	4.57 ± 1.17	8.39 ± 0.28^b^	139.50 ± 4.37^b^	41.47 ± 1.14^b^	49.10 ± 0.14	17.48 ± 0.12	342.33 ± 2.88	13.95 ± 0.85	30.72 ± 1.59^B^	121.33 ± 27.38	6.27 ± 0.16	15.40 ± 0.06	0.07 ± 0.01
T-1.54 mg/kg PAE	3.69 ± 1.15	8.18 ± 0.05	139.00 ± 4.20^a^	41.52 ± 0.58^b^	50.07 ± 0.27	16.97 ± 0.21	340.83 ± 5.91	13.61 ± 0.08	28.13 ± 0.20	145.83 ± 24.29	6.05 ± 0.14	15.58 ± 0.12	0.08 ± 0.02
T-3.08 mg/kg PAE	3.32 ± 1.09	8.18 ± 0.12	131.33 ± 2.07	39.51 ± 1.28	50.40 ± 0.38	17.12 ± 0.10	340.67 ± 1.21	13.90 ± 0.15	28.90 ± 0.36	145.50 ± 56.84	6.15 ± 0.21	15.12 ± 0.08	0.09 ± 0.03
T-20 mg/kg FCZ	3.58 ± 0.83	7.76 ± 0.18	130.50 ± 1.76	38.62 ± 0.57	50.40 ± 0.26	17.15 ± 0.14	340.00 ± 0.89	13.82 ± 0.26	28.63 ± 0.38	141.17 ± 47.60	6.27 ± 0.39	15.25 ± 0.23	0.09 ± 0.03

*^*a*^means compared with WT group *p* < 0.05, ^*b*^means compared with WT group *p* < 0.01, and ${}^{c}$means compared with WT group *p* < 0.01.*

### PAE Reduced Amounts of Immune Cells and Inflammatory Cytokines in the Vagina

**FIGURE 5:**
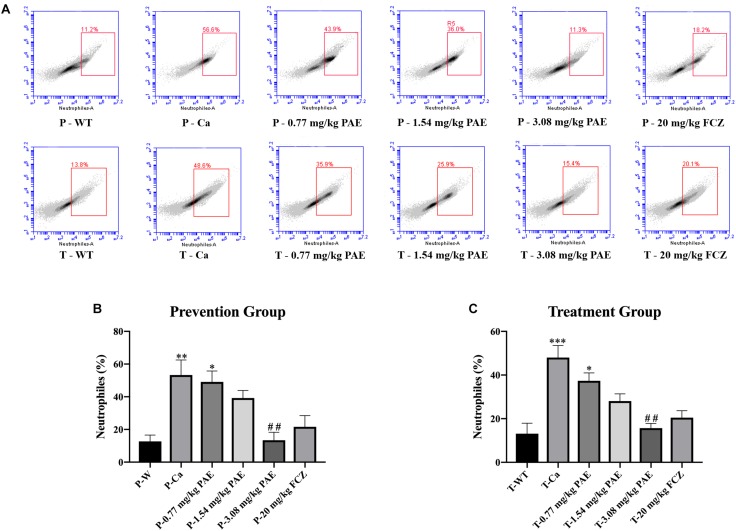
The neutrophil populations in vaginal tissue. **(A)** The number of neutrophils in the mouse vaginas was detected by flow cytometry in both the prevention group and the treatment group. **(B,C)** Statistical analysis of neutrophils in the prevention and treatment group. ^*^ means *p* < 0.05, ^∗∗^ means *p* < 0.01, ^∗∗∗^ means *p* < 0.001 compared with the WT group, ^##^ means *p* < 0.01 compared with the Ca group.

**FIGURE 6:**
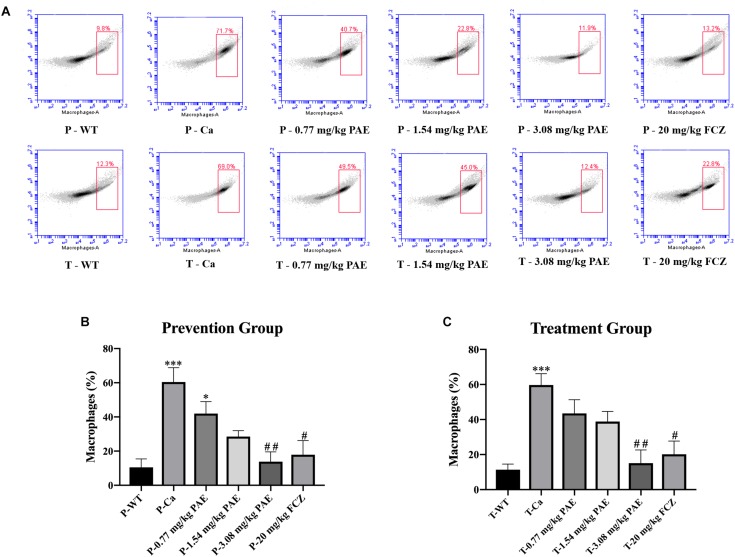
The macrophage populations in vaginal tissue. **(A)** The number of macrophages in the mouse vaginas was detected by flow cytometry in both the prevention group and the treatment group. **(B,C)** Statistical analysis of the macrophages in the prevention and treatment group. ^*^ means *p* < 0.05, ^∗∗∗^ means *p* < 0.001 compared with the WT group. ^#^ means *p* < 0.05, ^##^ means *p* < 0.01 compared with the Ca group.

**FIGURE 7:**
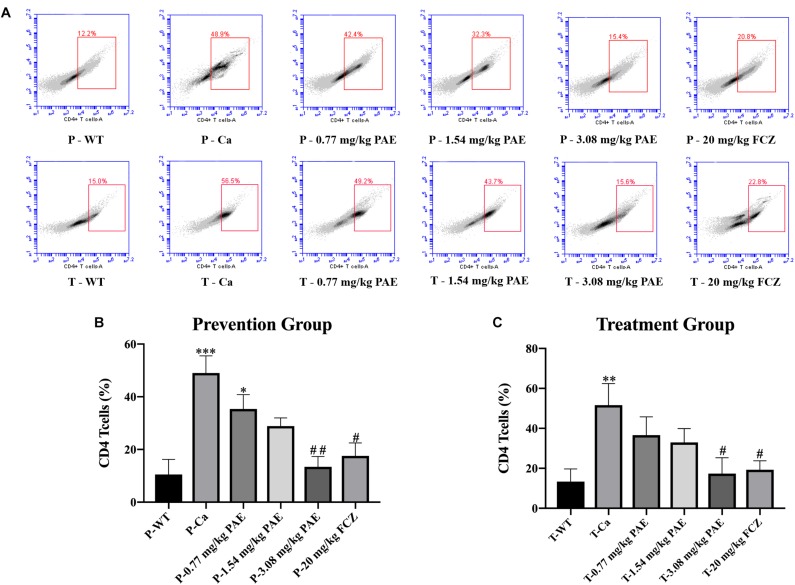
The CD4^+^ T cell populations in vaginal tissue. **(A)** The number of CD4^+^ T cells in mice vagina was detected by flow cytometry in the prevention group and the treatment group. **(B,C)** Statistical analysis of neutrophils in the prevention and treatment group. ^*^ means *p* < 0.05, ^∗∗^ means *p* < 0.01, ^∗∗∗^ means *p* < 0.001 compared with the WT group, ^#^ means *p* < 0.05, ^##^ means *p* < 0.01 compared with the Ca group.

**FIGURE 8:**
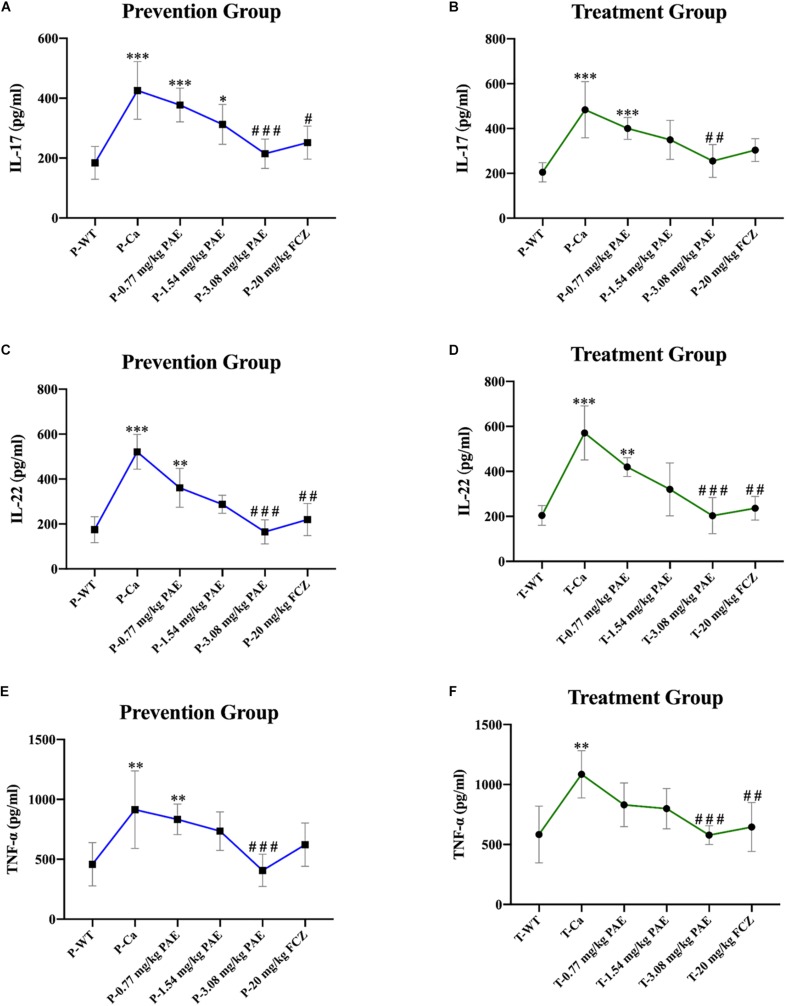
Pro-inflammatory cytokines IL-17, IL-22, and TNF-α detected in mouse vaginal tissue. **(A,B)** Shows elevation of IL-17 concentration detected by ELISA in both the prevention and treatment groups. ^*^ means *p* < 0.05, ^∗∗∗^ means *p* < 0.001, compared with the WT group, ^#^ means *p* < 0.05, ^###^ means *p* < 0.001 compared with the Ca group. **(C,D)** Elevation of IL-22 concentration detected by ELISA in the prevention and treatment groups. ^∗∗^ means *p* < 0.01, ^∗∗∗^ means *p* < 0.001, compared with the WT group, ^##^ means *p* < 0.01, ^###^ means *p* < 0.001 compared with the Ca group. **(E,F)** Elevation of TNF-α concentration detected by ELISA in both the prevention and treatment groups. ^∗∗^ means *p* < 0.01, ^∗∗∗^ means *p* < 0.001 compared with the WT group, ^##^ means *p* < 0.01, ^###^ means *p* < 0.001 compared with the Ca group.

The IL-17 and IL-22 responses are associated with the mycelial ability of *C. albicans*, which stimulates the neutrophils, macrophages, and T cells, and thus elicits a host-innate immune response (De Luca et al., 2013; Yu et al., 2018). We detected immune cells and related inflammatory cytokines in the vaginal mucosa by flow cytometry and ELISA (Figures 5–8). The numbers of neutrophils, macrophages, and CD4 T cells in the vagina were significantly increased after infection with *C. albicans*. When the mice were vaccinated with PAE, the levels of neutrophils, macrophages and CD4 T cells decreased, and there was no statistically significant difference between the 3.08 mg/kg PAE and the 20 mg/kg FCZ mice (Figures 5B, 6B, 7B). The same trend was also evident in the vaginal candidiasis-associated inflammatory cytokines, IL-17, IL-22, and TNF-α (Figure 8B). In the prevention group, we found that PAE reduced the number of immune cells and inflammatory cytokines in the vagina slightly more than in the treatment group (Figures 5A, 6A, 7A, 8A). In addition, high doses of PAE (3.08 mg/kg) were shown to be comparable to fluconazole in prevention and treatment.

### PAE Increased the Expression of E-Cadherin

**FIGURE 9:**
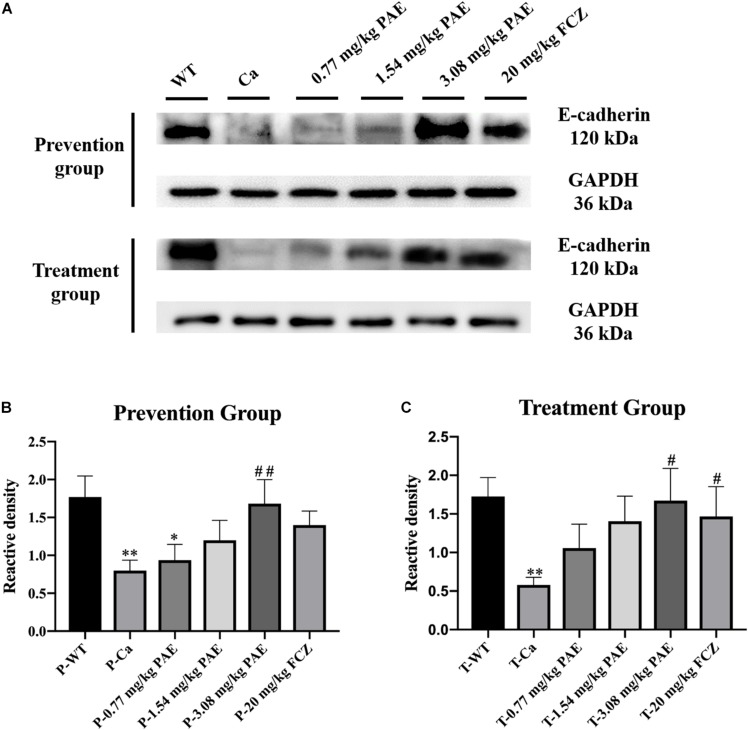
The detection of E-cadherin expression levels in vaginal tissue. **(A)** Western Blot analysis of E-cadherin expression in vaginal tissue. **(B)** Density assay of E-cadherin in prevention group. ^*^ means *p* < 0.05, ^∗∗^ means *p* < 0.01, compared with the WT group, ^##^ means *p* < 0.01 compared with the Ca group. **(C)** Density assay of E-cadherin in treatment group. ^∗∗^ means *p* < 0.01 compared with the WT group, ^##^ means *p* < 0.05 compared with the Ca group.

Previous researchers have indicated that proteolytic degradation of E-cadherin at the adhesion junction impairs the epithelial barrier function and facilitates *C. albicans* infection (Frank and Hostetter, 2007; Villar et al., 2007). In order to explore the mechanism of PAE prevention and treatment with vaginal candidiasis, we analyzed the expression of E-cadherin by the Western Blot method. We found that *C. albicans* inhibited the expression of E-cadherin. As shown in Figure 9, the expression of E-cadherin in the Ca group was significantly lower than that in the WT group. After prevention and treatment with PAE, the expression of E-cadherin was restored.

## Discussion

Vaginal candidiasis is a common gynecological disease; the clinical manifestations of vaginal candidiasis includes pruritus, profuse leucorrhea, burning pain, redness, and even restless sleep due to irritation of the vulva and vaginal mucosa (Peters et al., 2014). In the current study, we identified the excellent prevention and treatment effects of PAE in the murine model of VVC, and we identified the mechanism by which PAE functions against the inflammation in the vagina caused by *C. albicans*.

In the latest research, monoterpenoid PAE has been demonstrated to possess barely any genotoxic properties, and it poses no hidden danger for humans when used as a condiment in daily life. It may, therefore, be utilized as an alternative medicine in the management of inflammatory diseases or disorders (Hobbs et al., 2016; Fuyuno et al., 2018). Therefore, in this study, we verified the assumption that PAE decreases the WBC count to remit the inflammatory response in vaginal candidiasis. Furthermore, PAE reduced the concentration of immune cells, which had been abnormally elevated due to the *C. albicans.* These included the CD4^+^ T cells, the neutrophils, and the macrophages. Moreover, proinflammatory cytokines TNF-α, IL-17, and IL-22 all dropped back to a normal level after treatment by PAE. These data are consistent with a previous report in which innate immunity was identified as playing a crucial role in the regulation of vaginitis (Fidel et al., 2004).

The mouse model is an appropriate tool for research into pathogen identification and host defense in relation to fungal infections (Wang L. et al., 2016). In this research, our murine model of VVC was established with the support of estrogen, a hormone that is propitious to colonization by *C. albicans* (Figure 1A). Obvious *C. albicans* hyphae emerged in the vaginal lavage 1 day after invasion, and lots of robust hyphae appeared in the Ca group (Figure 1B). These data indicate that estrogen is an effective precipitator of VVC. Peters et al. (2014) indicate that the vaginal epithelium keratinizes and cornifies, resulting in epithelial thickening and ultimately sloughing off during menstruation. Another report demonstrates that women are more susceptible to VVC during the gestation period because of strong estrogenic activity during the menstrual cycle. Furthermore, vaginitis rarely occurs in low estrogen producing women, such as preadolescent girls and postmenopausal women (Hong et al., 2014). For this reason, a murine model of VVC was established to monitor the effect of PAE in VVC. On the last day of PAE treatment, we sacrificed the mice and isolated the vagina, uterus, fallopian tubes and ovaries from each. It became clear that the Ca group exhibited obvious swelling and inflammation. In contrast, the uterus in the 3.08 mg/kg PAE treatment group demonstrated the same uterus configuration as the WT group (Figures 1E,F).

Polymorphonuclear neutrophils (PMNs) have long been recognized as short-lived effector cells that have the ability to phagocytose pathogens and mediate tissue damage, thereby providing a first line of defense against invading pathogens (Nathan, 2006). In a large research project, which utilized female volunteers willing to be challenged with live *C. albicans*, the recruitment of PMNs into the vagina was demonstrated to be associated with vaginitis symptoms (Fidel et al., 2004). In our research, the large number of PMNs occurring in the vaginal canals of the Ca group, and the number of PMNs in the mouse vaginas, gradually decreased to normal levels with increased doses of PAE. Our H&E staining results also demonstrated that in tissues where PMNs were abundant, the integrity of the tissue structure was seriously damaged, including the keratin and squamous epithelium (Figure 4). These results verified the fact that, due to their powerful cytotoxicity, PMNs play a crucial role in inflammatory responses. Furthermore, when the release of PMNs is uncontrolled, the surrounding tissues may be destroyed by proteases, PMN-mediated ROS, and PMNs, all of which frequently occur in inflammatory disease (Cassatella et al., 2009). Our hematology results proved that WBC counts are elevated due to infection by *C. albicans*, while the WBC levels in PAE treated mice showed a tendency to decline. These results confirmed the anti-inflammatory effect of PAE and are consistent with recent reports (Uemura et al., 2018).

Neutrophils are a type of cell in the PMNs, and they are also vital in resisting bacterial and fungal invasion (Fan and Ley, 2017). The neutrophils are attracted to the sites of cell injury in diverse tissues, where they generate in dense swarms. The powerful weapon of the neutrophils is that they are resistant to pathogens and can cause a large amount of collateral damage, further extending immune cell activation, loss of functional tissue, and eventual organ dysfunction (Brandes et al., 2013). Similar results were also evident in our results, with the HE-staining and flow cytometry results showing that colonization by *C. albicans* recruited a large number of neutrophils into the vagina, but after treatment with PAE, the neutrophils in the tissue decreased significantly, accompanied by the gradual recovery of the tissue integrity (Figures 4, 5). These results are consistent with a previous report that indicated that inflammation is not always beneficial to the host (Uderhardt et al., 2019). Another key leukocyte in the inflammatory process is the monocyte, which moves from the blood into the tissues and matures into macrophages over a period of 2–3 days (Wang and Kubes, 2016). Our research discovered that *C. albicans* introduced a large number of macrophages, which assembled in the vagina. These results are consistent with those of a previous study (Yu et al., 2018). CD4^+^ T cells also play an important role in adjusting the immune response against self and foreign pathogens and in keeping immunological homeostasis. Naive CD4^+^ T cells can transform to create an additional T cell subset, Th17, which has been reported to possess the ability to produce IL-17A, IL-17F, IL-22, and CCL20 (Malefyt Rde, 2009; Roncarolo et al., 2018). In this study, CD4^+^ T cells also showed a steep rise in the Ca group, while in the PAE treatment group, the CD4^+^ T cells declined to nearly normal levels (Figure 7). In addition, TNF-α has been reported to be secreted by macrophages (Shin et al., 2017). Therefore, we next detected the related inflammatory cytokines expression in the vagina, which included IL-17, IL-22 and TNF-α.

IL-17 and IL-22 both play a key role in protective immunity, resisting *C. albicans* invasions, including both oral infections and vaginal infections (Pietrella et al., 2011; De Luca et al., 2013). In this study, we detected that IL-17 and IL-22 were enhanced after the invasion by *C. albicans*. However, the excessive expression of the inflammatory cytokines worsens with disease and finally prevents pathogen eradication (Romani et al., 2008). As in Romani et al.’s study, we found that the content of IL-17 and IL-22 in the PAE treated mice showed a gradual downward trend, and the interleukin levels in the 3.08 mg/kg PAE treated mice recovered to a nearly normal concentration in the vagina. These results were also consistent with our WBC, H&E staining and neutrophils data, and Matsuzaki and Umemura (2007) verifying that the activity of IL-17 against extracellular microbes also includes neutrophil recruitment to the invasion position. TNF-α, which is a blasting fuse of NF-κB or RIPK1 kinase-dependent cell death, is a pleiotropic cytokine that plays an important role in the mammalian inflammatory response, and many inflammatory pathologies are now recognized to be actuated by aberrant TNF-mediated cell deaths (Cook et al., 2018). In the murine model of vaginitis, the concentration of TNF-α in the Ca group increased to nearly double the concentration in the WT group, with the PAE evidencing the ability to reduce the TNF-α concentration to normal levels (Figures 8E,F). We therefore speculated that PAE may inhibit the NF-κB transfer from the cytoplasm to the nuclear material through its ability to decrease the expression of TNF-α in VVC, and further inhibits the release of pro-inflammatory cytokine IL-1β to reduce the inflammation in the tissues. The latest research reports that TNF and ROS impact on each other in a positive feedback loop (Blaser et al., 2016). PAE has also been reported to attenuate CUMS-introduced depressive-like behaviors by regulating NLRP3 (Song et al., 2018). In our study, PAE exhibited prominent anti-inflammatory effects, but its full mechanism still needs to be explored.

Finally, we detected E-cadherin expression in the vagina. The proteolytic degradation of E-cadherin has been reported to damage and compromise the epithelial barrier function in the adherens junctions, and this promotes invasion *of C. albicans* in oropharyngeal candidiasis (Villar et al., 2007). In our research, *C. albicans* invasion also inhibited the expression of E-cadherin, but PAE was able to solve this problem and the E-cadherin activity was recovered in the epithelium to protect the tissues against the *C. albicans* invasion. Recent research indicates that E-cadherin degradation is also associated with the transmigration of neutrophils and infection of tissues *by C. albicans*, and these results are also consistent with our data (Xu et al., 2016). PAE reduces the quantity of neutrophils and *C. albicans* in vaginal tissue and enables E-cadherin activity to recover to further protect the tissue.

In summary, this work has shown that PAE is effective in both the prevention and treatment of vaginal candidiasis. If a lotion containing PAE is used to wash the vagina, the user may suffer less than others. The success of this murine study using an animal model for vaginal candidiasis provides a new strategy for the investigation and treatment of vaginal candidiasis and explores the mechanism by which PAE functions against *C. albicans* in the murine model of VVC. This study provides a theoretical basis for PAE to become a clinical antifungal agent in the future.

## Ethics Statement

This study was carried out in accordance with the recommendations of the International Association on the use and care of laboratory animals. The protocol was approved by the Ethics Committee of the Jiangsu Normal University.

## Author Contributions

JT and ZZF designed the experiments. SQ, LC, HT, LW, FW, ZW, and LX performed the experiments. JL, ZJF, and HJ analyzed the data. SQ, LC, and HT drafted the manuscript. All authors read and approved the final manuscript.

## Conflict of Interest Statement

The authors declare that the research was conducted in the absence of any commercial or financial relationships that could be construed as a potential conflict of interest.
